# The Cost of Provisions in Institutions.—XIV

**Published:** 1904-08-06

**Authors:** 


### HOSPITAL ADMINISTRATION.

### CONSTRUCTION AND ECONOMICS.

### THE COST OF PROVISIONS IN INSTITUTIONS.—XIV.

BY THE AUTHOR OF "HOSPITAL EXPENDITURE—THE COMMISSARIAT."

(*Continued from page* 300.)

#### THE PRICE OF MUTTON.

THE last ten years have brought about a great change in the attitude of the householder towards "foreign meat." It is not very usual at the present day to meet with people who still believe anæmia and other diseases to be the natural outcome of eating any but British produce, but such alarmists were common enough some years ago, and in large institutions fought desperately against the introduction of frozen meat. The use of New Zealand mutton was often a jealously guarded secret; and the long-standing prejudice against it has only gradually melted away before the indisputable fact that the objectors are exposed to the practical annoyance of being unable to distinguish it from English meat when set before them on the table.

There is a good deal of difference in the price of foreign mutton. The normal variation in price may be understood from the following table, from which it will be seen that the all-round price swings between 3½d. and 5½d. per lb.

*Mutton (Frozen.)*

(Compiled from the *Annual Reports* of the Asylums Committee of the London County Council.)

| Year | Price per stone (14 lb.) | | | | | |
|---|---|---|---|---|---|---|
| | Banstead | | Cane Hill | | Claybury | |
| | March | October | March | October | March | October |
| | s. d. | s. d. | s. d. | s. d. | s. d. | s. d. |
| 1895 ... ... | 4 10 | 5 10 | 4 10 | 5 10 | 4 10 | 5 10 |
| 1896 ... ... | 4 6 | 4 1 | 4 6 | 4 1 | 4 6 | 4 1 |
| 1897 ... ... | 4 5 | 4 6 | 4 0 | 4 2 | 4 0 | 4 6 |
| 1898 ... ... | 4 6 | 4 11 | 4 6 | 4 11 | 4 6 | 4 6 |
| 1899 ... ... | 4 9 | 4 11 | 4 6 | 4 11 | 4 11 | 4 11 |
| 1900 ... ... | 5 8 | 6 5 | 5 8 | 6 5 | 5 8 | 6 5 |
| 1901 ... ... | 6 3 | 4 2 | 6 3 | 4 2 | 6 3 | 4 2 |
| 1902 ... ... | 6 2 | 5 10 | 6 0 | 5 10 | 6 0 | 5 10 |

*Note.*—The Contracts made in March run for seven months (April to October), and those in October for five months (November to March).

The retail price (1902) starts at 2½d. for breasts, and rises to 7d. per lb. for legs, with a difference of ½d. for certain joints, between the March and September prices. This is for New Zealand Mutton in London.

Referring to market prices for the whole sheep at Smithfield, it will be found that under the head of "Frozen Mutton" the buyer has now the choice of three varieties—New Zealand, Australian, and River Plate. Dutch mutton brought over alive ranks as "English killed."

As regards price and quality New Zealand still easily holds the first rank for frozen mutton. The wholesale winter price works out at from 3¼d. to 4¼d., all according to size and quality. Australian mutton, the whole sheep, 3d. lb.; River Plate, 3¼d. The contractor, who undertakes the supply of frozen meat to institutions, will, in all probability, draw upon all these supplies. The questions to be considered are whether the authorities appreciate the distinctions in price, and whether they have in their service an official who understands how to distinguish the one kind of meat from the other. Owing to the prevalent ignorance relating to foreign supplies of meat Australian and River Plate mutton are very commonly indeed supplied at the rate of the best New Zealand, to the complete satisfaction of all concerned in the transaction. The attitude of the busy officials responsible for the commissariat, is very generally that, so long as the quality is fairly good, and the price fairly low, nothing more need be expected. This may do very well for the private household, but in the great institution the urgent problem is to get the best possible quality at the lowest possible price; and nothing short of this should satisfy.

If the best New Zealand meat then is contracted for, there should be some one to receive it who can tell it from lower-priced frozen meat. If it is found by experience that the consumers do not know the difference when the lower-priced mutton is substituted let the contract be framed for this class of meat, and because the price is low let stringent precautions be taken in the weighing and ceaseless vigilance be exercised to prevent the inclusion of unsound mutton from other sources. A common defect of this class of mutton is sourness at the edges, and unless carefully hung it will easily take a stale flavour. But, indeed, the same may be said of any kind of meat.

The more closely the meat supply to large institutions is considered the more clear it becomes that the best results, both as regards economy and the comfort of the household, are to be obtained by purchasing by the whole sheep. In those institutions where this is done one of the porters is engaged with the necessary qualifications for cutting the joints, and large premises are not found to be indispensable. A light and airy cellar is used, and a good larder is a *sine quâ non.* Under such a system it is possible to victual the hospital with English mutton of the second grade or with Dutch mutton of excellent quality at from 4½d. to 5d. per lb., and it has been shown that New Zealand and River Plate frozen mutton can be had at the all-round price of from 3d. to 4d. per lb. Vigilant care is needed in the reception of the meat. Moreover, the housekeeper finds her cares are no longer confined to the monotonous task of apportioning to the different tables so many legs of mutton and so many rounds of beef. Her ingenuity will be a little taxed to turn every part of the animal to good account, and the cook will be called on for a corresponding effort in the making of savoury dishes. In the last resort the stock-pot for patients' broth will prove a ready receptacle for odds and ends and obviate any risk of waste.

This is not chimerical. It can be done and is done. We might almost say that to produce a minimum expenditure it must be done.

The hospital or institution household is a very composite one, and must of necessity contain a large proportion of working men and women who are entirely unaccustomed to be fed on slices of roast leg of mutton. They would not only be perfectly contented with haricot, Irish stew, camp pie, and other dishes of the kind; they would in all probability greatly prefer such fare. In fact, the monotony of the stock hospital joints, alternately roast and boiled, weighs on every inmate, from the highest to the lowest, even when the meat is well chosen, well hung, well cooked, and well carved. When any of these four points gives way, when the meat is coarse, too fresh, over or under cooked, or wrenched off the joint in jagged lumps, its appearance on the plates is discouraging to the most eager appetite. Probably a cheap, well-made stew would be welcomed as a delightful variety at every table.

When all has been done that can be done in the steward's department to ensure the delivery of good, sound meat to the institution, the part played by the cook is, after all, by far the most important.

Take the most expensive meat you can get at the highest-priced butcher, and an ignorant cook will make it uneatable by a turn of her hand. Take, on the other hand, the cheapest frozen meat, and, if it be perfectly sound and well hung, a clever cook will produce from it a dish fit for an epicure.

The moral then is that for the carrying out of any system of minimum expenditure, a well-paid and well-taught cook is an absolute necessity. To pay an extra £10 a year and secure a cook who really understands her business may result in saving ten times that amount in the end. Everyone in the institution is working at high pressure, and it is essential that the food set before them shall be light, nourishing, and easily digested. The ordinary plain cook as she figures in institution housekeeping has no conception about food values or meat prices, or the effect on the materials she uses, produced by the processes to which she subjects them. And the ordinary matron has, it may be feared, even less knowledge of such things than the ordinary cook.

Contracting for the whole sheep, and exercising ordinary care in its reception and preparation, a saving of from 1d. to 2d. per lb. in the cost of mutton is effected in many institutions where the commissariat is beyond reproach. Something like 30,000 lb. of mutton will be consumed in a year in even a moderate-sized hospital, and it will not be denied that the £250 which might often be saved over this one article would be worth taking a little trouble to secure.

In conclusion we give the Smithfield Market quotations for mutton, July 23rd, 1904.

*Per Stone of 8 lb.*

| | s. | d. | | s. | d. |
|---|---|---|---|---|---|
| Scotch wethers ... ... ... ... | 5 | 4 | to | 5 | 6 |
| " ewes ... ... ... ... ... | 3 | 6 | to | 3 | 8 |
| English wethers (light) ... ... | 4 | 8 | to | 5 | 0 |
| " " (heavy) ... ... | 4 | 2 | to | 4 | 4 |
| English ewes (light) ... ... ... | 3 | 4 | to | 3 | 8 |
| " " (heavy) ... ... | 3 | 0 | to | 3 | 2 |
| New Zealand Canterbury ... ... | 2 | 10 | to | 3 | 0 |
| " " Wellington ... ... | 2 | 8 | to | 2 | 10 |
| River Plate ... ... ... ... | 2 | 5 | to | 2 | 8 |

The wholesale all-round price at the present time ranges, it will be seen, from 8½d. to 4½d. for English or Scotch mutton, and from 4½d. to 3½d. for frozen mutton.

It will be seen that there is as much distinction according to quality in English killed mutton between the best quality and the coarse-fibred meat, as there is between English and foreign. And as all these variations in price take their rise from well understood gradations in the flavour and quality of the meat when sent to table, it would appear that better results may be expected from good frozen meat than from the old ewes and "heavy" mutton which the contractor is compelled to furnish when he is limited to English supplies at a low price.

